# The complete mitogenome and phylogenetic analysis of Japanese firefly ‘Genji Botaru’ *Luciola cruciata* (Coleoptera: Lampyridae)

**DOI:** 10.1080/23802359.2017.1365641

**Published:** 2017-08-16

**Authors:** Juri Maeda, Dai-ichiro Kato, Kazunari Arima, Yuji Ito, Atsushi Toyoda, Hideki Noguchi

**Affiliations:** a Department of Chemistry and Bioscience, Graduate School of Science and Engineering, Kagoshima University, Kagoshima, Japan;; b Advanced Genomics Center, National Institute of Genetics, Mishima, Japan;; c Center for Genome Informatics, Joint Support-Center for Data Science Research, Research Organization of Information and Systems, Mishima, Japan

**Keywords:** *Luciola cruciata*, firefly, Lampyridae, mitochondrial genome, Coleoptera

## Abstract

We performed mitogenome analysis of Japanese firefly *Luciola cruciata* (Coleoptera: Lampyridae), which is unique species in Japan. It is classified into six haplotypes based on the difference of COII sequence on mitochondrial DNA. The complete mitogenome sequence of Tohoku group has been registered so far, we newly analysed West Japan groups which belong to different haplotype. The total length of analysed mitogenome was 15,990 bp, being one base longer than the case of Tohoku’s firefly. The base substitution was found at 273 positions over the whole mitochondrial sequence, while amino acid substitution accompanying it was observed at only 11 positions.

The Japanese firefly *Luciola cruciata* (Coleoptera: Lampyridae) is one of the most emblematic insect on early summer, is widely distributed throughout Japan except for Hokkaido. Its fantastic light has been attracted people, thus Japanese have loved it from ancient times. This bioluminescence reaction is achieved by the oxidation of the substrate d-luciferin to oxyluciferin by the enzyme firefly luciferase, and widely applied in academic research as well as various biochemical analysis such as imaging tools (Contag and Bachmann 2002), ATP measurement reagents (Kimmich et al. 1975) and NGS reading method (Reuter et al. 2015).

In Japan, *L. cruciata* is known to be divided into roughly two groups depending on the difference of emission cycles and oviposition manners (Suzuki et al. 1996). Two groups could be further divided into six haplotypes based on the difference of COII sequence in mitogenome (Suzuki et al. 2004). The mitogenome sequence of Tohoku firefly, which belongs to a type of four second emission cycles, was already registered in 2013 as GenBank No. AB849456 (Amano et al. 2013). In this paper, we analysed the mitogenome of another *L. cruciata* which is classified into a type of two second emission cycles captured in West Japan area.

The specimens of adult male firefly used in this study were collected from Yamabe county, Nara prefecture, Japan (34°38′06.1″N, 136°02′19.4″E) and was stored in our laboratory at −80 °C. The whole body was homogenized by ball mill and total genomic DNA was extracted using QIAGEN Blood & Cell DNA Min Kit (Hilden, Germany). The sequencing was performed by Illumina Hiseq. Assembling process was carried out by the de novo sequence assembler Platanus. We used a software Bioedit v7.2.5 to annotate cording regions in reference to the registered data (AB849456). The annotated genome sequence was submitted to GenBank with accession number LC306677.

The total length of analysed mitogenome was 15,990 bp, which was one base longer than the case of Tohoku’s firefly by insertion of cytosine base at the 179th position on AT-rich region. The single base substitution occurred on 273 positions over the whole mitogenome, thus the base composition was a little different; A (43.20 → 43.01%), T (32.53 → 32.46%), G (9.33 → 9.39%), C (14.95 → 15.13%) (Tohoku’s sequence (AB849456) → This work). Among their mutation, the 226 sites of base substitution were observed in the CDS region, while almost base substitutions result in the silent mutations and amino acid exchanges occurred at only 11 positions on six CDSs (ND2, COII, COIII, ATP8, ND4, and ND6). No amino acid substitutions were found in seven CDSs (COI, ATP6, ND3, ND5, ND4L, CytB, and ND1).

We constructed the neighbour-joining tree by MEGA6 with 1000 bootstrap replicates using *Tribolium castaneum* (AJ312413) and 10 kinds of Lampyridae *L. cruciata* (AB849456, LC306677), *L. lateralis* (LC306678), *L. substriata* (KP313820), *Aquatica leii* (KF667531), *Aquatica ficta* (KX758085), *Aquatica wuhana* (KX758086), *Asymmetricata circumdata* (KX229747), *Pyrocoelia rufa* (AF452048), and *Bicellonychia lividipennis* (KJ922151) (Tribolium Genome Sequencing Consortium 2008) (Figure 1). The bootstrap showed high values at all nodes. The result indicated that *L. cruciata* is closest to *A. leii*, *A. ficta*, *A. wuhana*, and *L. lateralis*.

**Figure 1.**
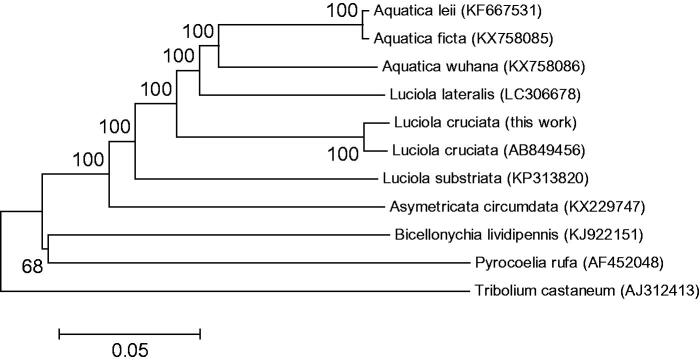
The phylogeny using complete mitochondrial genome sequence of *L. cruciata*, *T. castaneum*, and seven firefly species. The complete mitogenome of each species was obtained from GenBank and the phylogenetic tree was constructed by MEGA6 using neighbour-joining method with 1000 bootstrap replicates.
